# Diagnostic and prognostic significance of miR-320a-3p in patients with chronic heart failure

**DOI:** 10.1186/s12872-024-03966-0

**Published:** 2024-06-17

**Authors:** Qing Han, Li Zhang, Ran Liao

**Affiliations:** Department of Cardiovascular Medicine, Jiujiang City Key Laboratory of Cell Therapy, Jiujiang NO.1 People’s Hospital, No. 48, Taling South Road, Xunyang District, Jiujiang, 332000 China

**Keywords:** Chronic heart failure, MiR-320a-3p, Diagnosis, Prognosis

## Abstract

**Aim:**

The purpose of this study was to investigate the diagnostic and prognostic value of miR-320a-3p in chronic heart failure (CHF).

**Methods:**

A total of 103 patients with CHF and 95 healthy controls were included in the study population. The expression level of serum miR-320a-3p was detected by qRT-PCR. The diagnostic effect of miR-320a-3p on CHF was evaluated by receiver operating characteristic curve. Kaplan-Meier curve and Cox regression were used to analyze the risk factors for 4-year prognosis of CHF patients. Bioinformatics analysis was used to analyze the possible target genes of miR-320a-3p and related signaling pathways.

**Results:**

Serum miR-320a-3p expression was increased in CHF patients, and the levels of BNP and LVEF were positively and negatively correlated with miR-320a-3p, respectively. The AUC value of ROC curve was 0.866, indicating that miR-320a-3p had high diagnostic accuracy for CHF. Survival curve and Cox analysis showed that high expression of miR-320a-3p was associated with poor prognosis in CHF patients, and age and miR-320a-3p were independent risk factors for prognosis in CHF patients. GO and KEGG analysis showed that the downstream target genes of miR-320a-3p were involved in biological processes such as cell adhesion, stem cell differentiation and neural development, and were enriched in mTOR, TNF, AMPK and other signaling pathways.

**Conclusions:**

miR-320a-3p increased abnormally in CHF and was related to the severity of CHF. miR-320a-3p has the potential to be a diagnostic and prognostic marker for CHF.

## Introduction

Chronic Heart failure (CHF) is a complex syndrome featured with abnormal changes in the structure and function of the heart caused by various causes, which hinders the ejection function and filling of the ventricle, and the cardiac output is insufficient to meet the needs of tissue metabolism [1, 2]. The clinical manifestations are congestion in pulmonary circulation and systemic circulation and insufficient perfusion of tissues and organs, mainly manifested as dyspnea, fatigue, limited physical activity and fluid retention. CHF is still a serious public health problem in the world [3]. It is estimated that by 2030, the incidence of CHF will increase by 25%, and the medical expenditure related to HF will more than double [4]. Diagnosis and risk stratification of CHF mainly rely on symptoms, signs, blood biomarkers, and cardiac ultrasound, where the potential value of blood biomarkers is greater and more complex. BNP has good diagnostic value, sensitivity and predictive value for CHF, but its disadvantage is that it is easily influenced by age, renal function and various diseases [5]. Therefore, it is imperative to find an index with good sensitivity and specificity for CHF diagnosis without increasing the cost.

MicroRNA (miRNA) is a non-coding RNA with a length of about 24 nucleotides, which plays a regulatory role by specifically binding to the 3’-UTR region of mRNA at the post-transcriptional level [6, 7]. MiRNAs with good stability have been detected in human blood and other body fluids, which are called circulating miRNAs. Since miRNA was discovered in blood in 2008, circulating miRNA has been found in blood, urine, tears, and other body fluids [8, 9]. Stable miRNAs can be detected in peripheral blood and can be rapidly detected, which makes it possible to become the potential biomarkers for clinical diagnosis and treatment guidance [10]. The study of miRNAs as markers has aroused great interest among researchers. There have been many advances in the study of miRNAs in heart disease, and identifying the functions of key miRNAs will help elucidate the pathogenesis of heart failure. One study suggested that miR-19b was declined in heart failure patients with dilated cardiomyopathy and aortic stenosis [11]. Another study showed that miR-125 b inhibited cardiomyocyte apoptosis by targeting BAK1, thus reducing the cardiac function damage in mice with heart failure [12]. With the development of more in-depth research, more and more miRNAs have begun to enter clinical trials and participate in the exploration of disease treatment. MiR-320a is located in 8p21.3 of chromosome [13]. Current studies on miR-320a-3p mainly focus on tumors and also involve some cardiovascular diseases. For example, Galeano et al. ‘s study revealed that the level of miR-320a-3p showed a rapid time-dependent increase in patients with ST-segment elevation myocardial infarction [14]. Marques et al. found abnormal expression of dozens of miRNAs, including miR-320a-3p, in arterial blood of patients with congestive heart failure [15]. Although miR-320a-3p has been found to be related to heart failure, the study of miRNA diagnosis and prognosis of CHF remains unclear.

This present study aims to measure the expression of miR-320a-3p in patients with CHF, evaluate the diagnostic and prognostic value of miR-320a-3p in CHF, and further explore the target genes of miR-320a-3p and its possible mechanism of action, providing a valuable basis for the exploration of biomarkers of CHF.

## Materials and methods

### Study population

A total of 103 patients diagnosed with chronic heart failure in Jiujiang NO.1 People’s Hospital were selected. In addition, 95 healthy people in the same period and excluded chronic heart failure were selected as the control group. The diagnosis of CHF follows the 2016 European Society of Cardiology (ESC) Guidelines for the Diagnosis and treatment of acute and chronic heart failure [16]. Patients have been diagnosed with CHF for at least 6 months. The exclusion criteria of the two groups were as follows: (1) patients with acute coronary syndrome, congenital heart disease, pulmonary heart disease, malignant tumor, etc.; (2) patients with liver and kidney failure; (3) stroke, acute cerebrovascular accident within six months. This project has been approved by the Ethics Committee of Jiujiang NO.1 People’s Hospital, and all subjects have signed written informed consent. All procedures in the program are carried out in strict accordance with the guidelines of the Declaration of Helsinki on human trial.

### Data collection

After enrollment, gender, age, body mass index (BMI), smoking history (smoking history is defined as current smoking, previous smoking, or never smoking), drinking history (drinking history is defined as drinking alcohol at least once a week for more than 3 months), total cholesterol (TC), triglyceride (TG), low-density lipoprotein cholesterol (LDL-C), high-density lipoprotein cholesterol (HDL-C), brain natriuretic peptide (BNP), left ventricular ejection fraction (LVEF), and New York heart association (NYHA). On the next day after enrollment, fasting venous blood of all subjects was collected for further experiment.

### Quantitative real‑time polymerase chain reaction (qRT‑PCR)

TRIzol reagent was used to extract total RNA from serum, and NanoDrop 2000 was used to determine the concentration and purity of RNA. RNA was reverse-transcribed by PrimeScript RT kit to synthesize cDNA, then qRT-PCR reaction was performed. The reaction system consisted of 10µL, including 1µL cDNA, 0.3µL forward and reverse primes, 5µL SYBR Premix Ex Taq, and 3.7µL H_2_O. The amplification procedure was as follows: pre-denaturation at 95℃ for 5 min, with 40 cycles of denaturation at 95℃ for 15s, annealing at 60℃ for 20s, extension at 72℃ for 15s. Using U6 as internal parameter, and the relative expression of miR-320a-3p was calculated by 2^−△△C^t method.

### Follow-up analysis

103 CHF patients received conventional clinical treatment for CHF, including angiotensin-converting enzyme inhibitors, beta-receptor antagonists, diuretics. Subsequently, according to the mean value of miR-320a-3p, the patients were divided into two groups, namely, high expression of miR-320a-3p group (*n* = 54) and low expression of miR-320a-3p group (*n* = 49). In addition, based on the average value of BNP, patients were divided into a group with high BNP expression (*n* = 53) and a group with low BNP expression (*n* = 50). Both groups were followed up for 4 years, mainly through telephone interviews. During the follow-up period, death was taken as the end event, and the death situation of the patients was counted for subsequent analysis.

### Target gene prediction of miR-320a-3p

In order to improve the scientific and reliability of target gene prediction, this study used TargetScan 8.0, miRBD and EVmiRNA databases to predict the target genes of miR-320a-3p, and presented the predicted target genes in the form of Venn diagram.

### GO and KEGG pathway enrichment analysis

The target genes predicted by the above three databases were further analyzed. GO analysis was performed using DAVID 6.7 software to determine molecular function (MF), cell composition (CC), and biological processes (BP) of target genes. The signal pathway of possible enrichment of target genes was analyzed by KEGG database (http://www.genome.jp/kegg/).

### Statistical analysis

SPSS 22.0 and GraphPad Prism 7.0 software were used for data analysis. Kolmogorov-Smirnov test was used to evaluate the normality of the data. Data conforming to the normal distribution were expressed as mean ± standard deviation (SD) and were compared by independent sample T-test or one-way ANOVA. Counting data was represented by n, and Chi-square test was used for comparison between groups. The diagnostic value of miR-320a-3p was evaluated by drawing the working characteristic curves of subjects. The Pearson correlation coefficient evaluated the association of BNP or LVEF with serum miR-320a-3p levels in patient group. Kaplan-Meier curve and Cox regression were used to evaluate the prognostic value of miR-320a-3p. *P* < 0.05 was defined as a significant difference.

## Results

### Comparison of baseline data

The comparison of baseline data and clinical indicators between the 95 control cases and 103 CHF patients is shown in Table 1. The results showed that there were no statistically significant differences in age, sex, BMI, smoking history, drinking history, hypertension history, diabetes history, and levels of TC, TG, LDL-C between the two groups (*P* > 0.05). In addition, HDL-C and LVEF levels in the CHF patients were significantly lower than those in the control group, while BNP levels were higher than that in the control group (*P* < 0.05). According to the principle of NYHA classification, 103 patients with CHF included 20 patients with NYHA-I, 35 patients with NYHA-II, 28 patients with NYHA-III and 20 patients with NYHA-IV.

**Table 1:** Comparison of baseline characteristics between CHF patients and healthy controls

Items	Control group (*n* = 95)	CHF group (*n* = 103)	*P*
Age (years)	65.72 ± 1.51	65.33 ± 1.53	0.148
Gender (male/female)	57/38	60/43	0.458
BMI (kg/m^2^)	23.47 ± 0.41	23.59 ± 0.39	0.120
TC (mmol/L)	4.37 ± 1.72	4.67 ± 1.58	0.580
TG (mmol/L)	1.26 ± 0.37	1.31 ± 0.36	0.299
LDL-C (mmol/L)	2.76 ± 0.29	2.81 ± 0.26	0.068
HDL-C (mmol/L)	1.29 ± 0.23	1.18 ± 0.24	0.017
BNP (pg/mL)	62.13 ± 16.78	769.86 ± 88.93	< 0.001
LVEF (%)	52.75 ± 5.94	33.23 ± 4.09	< 0.001
Smoking history (yes/no)	53/42	56/47	0.477
Drinking history (yes/no)	58/37	63/40	0.551
Hypertension (yes/no)	63/32	70/33	0.462
Diabetes (yes/no)	56/39	62/41	0.786
NYHA stage			
I	/	20	/
II	/	35	/
III	/	28	/
IV	/	20	/

Abbreviations: CHF, chronic heart failure; BMI, body mass index; TC, total cholesterol; TG, triglyceride; LDL-C, low-density lipoprotein cholesterol; HDL-C, high-density lipoprotein cholesterol; BNP, brain natriuretic peptide; LVEF, left ventricular ejection fraction; NYHA, New York heart association. *P* < 0.05 stands for significant difference

### The expression level and diagnostic value of miR-320a-3p in CHF

**Fig. 1:**
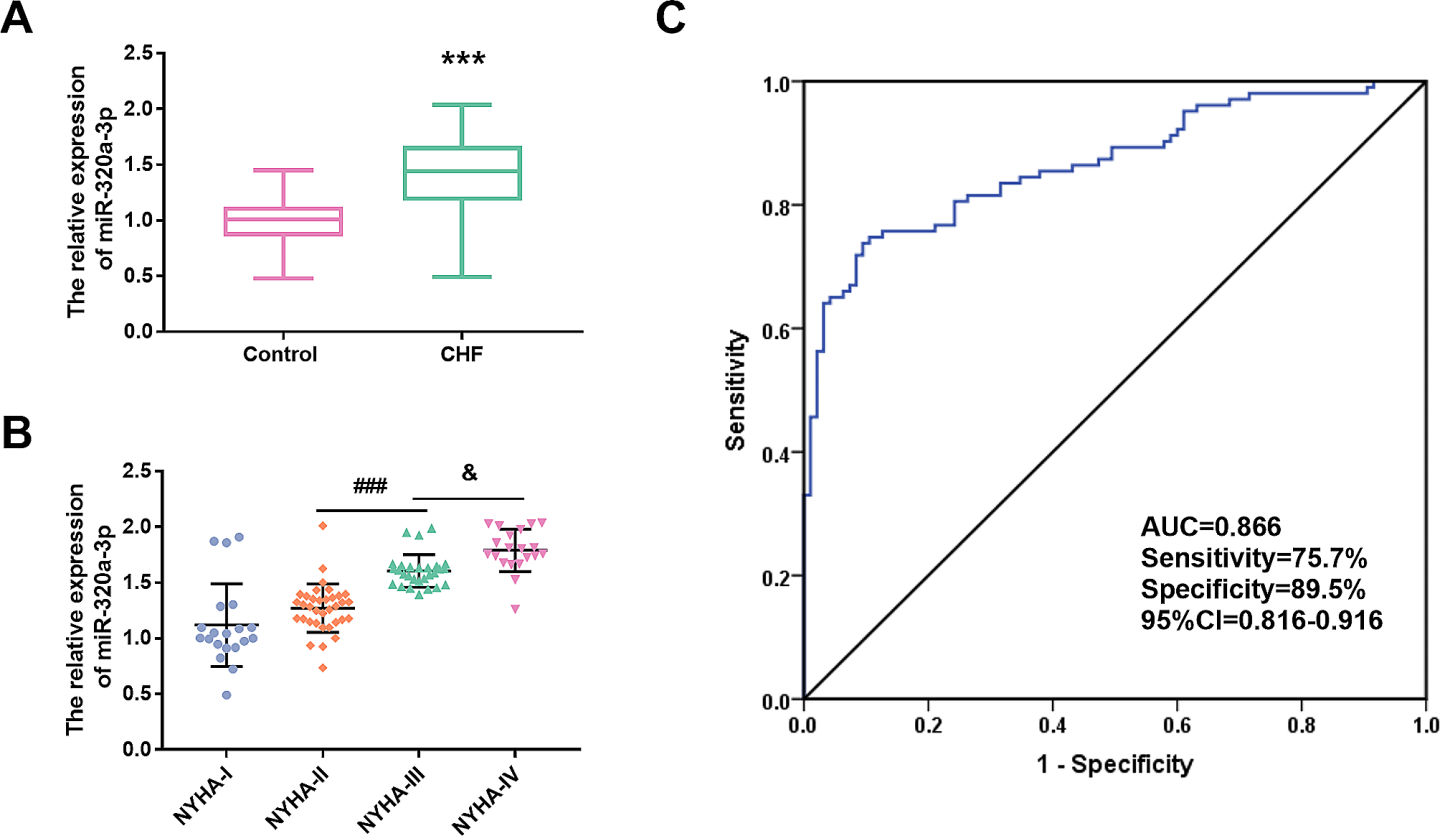
Expression level of miR-320a-3p and its diagnostic value. **(A)** miR-320a-3p expression was elevated in patients with chronic heart failure (CHF). **(B**) miR-320a-3p increased with the increase of NYHA classification. **(C)** ROC curve indicated that miR-320a-3p had diagnostic value in CHF. ^***^P < 0.001 vs. Control group. ^###^*P* < 0.001 vs. NYHA II group. ^&^*P* < 0.05 vs. NYHA III group

qRT-PCR results demonstrated that the serum level of miR-320a-3p in CHF group was significantly higher than that in the control group (Fig. 1A, *P* < 0.001). Further, results showed that the expression level of miR-320a-3p gradually upregulated with the improvement of NYHA grade. The serum miR-320a-3p in NYHA III patients was significantly higher than that in NYHA II patients, and the miR-320a-3p level in NYHA IV patients was further higher than that in NYHA III patients (Fig. 1B, *P* < 0.05), which preliminarily speculated that the level of miR-320a-3p may be related to the severity of CHF. In addition, based on the abnormal expression of miR-320a-3p in CHF, the ROC curve of miR-320a-3p was constructed in this study. Figure 1C showed that the curve has a high area under the curve (AUC), sensitivity and specificity, which are 0.866, 75.7% and 89.5%, respectively, indicating that miR-320a-3p as a diagnostic marker for CHF has a high diagnostic accuracy.

### Correlation analysis

**Fig. 2:**
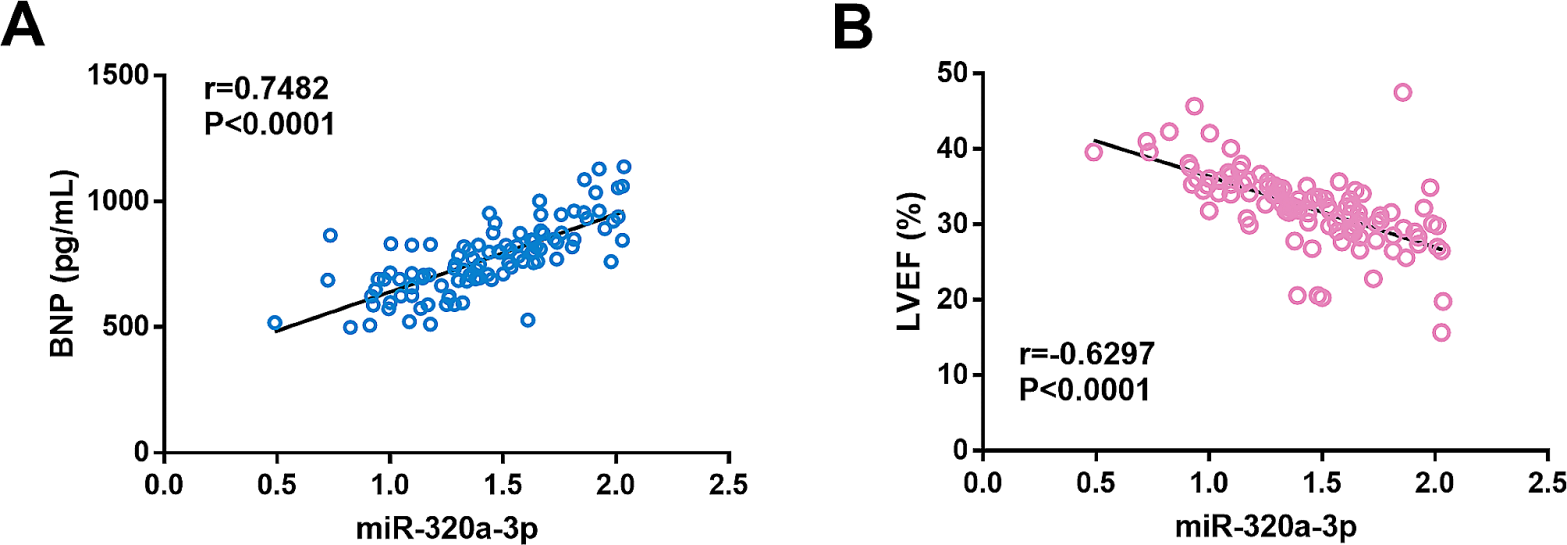
Correlation analysis. **(A)** Brain natriuretic peptide (BNP) is positively correlated with miR-320a-3p. **(B)** Left ventricular ejection fraction (LVEF) is negatively correlated with miR-320a-3p

In order to further explore the relationship between miR-320a-3p and CHF, Pearson method was used to evaluate the correlation between miR-320a-3p and BNP or LVEF. The results showed that BNP was positively correlated with the level of miR-320a-3p (Fig. 2A, *r* = 0.7482, *P* < 0.001), and LVEF was negatively correlated with miR-320a-3p (Fig. 2B, *r* = -0.6297, *P* < 0.001).

### Prognostic value analysis of miR-320a-3p

**Fig. 3:**
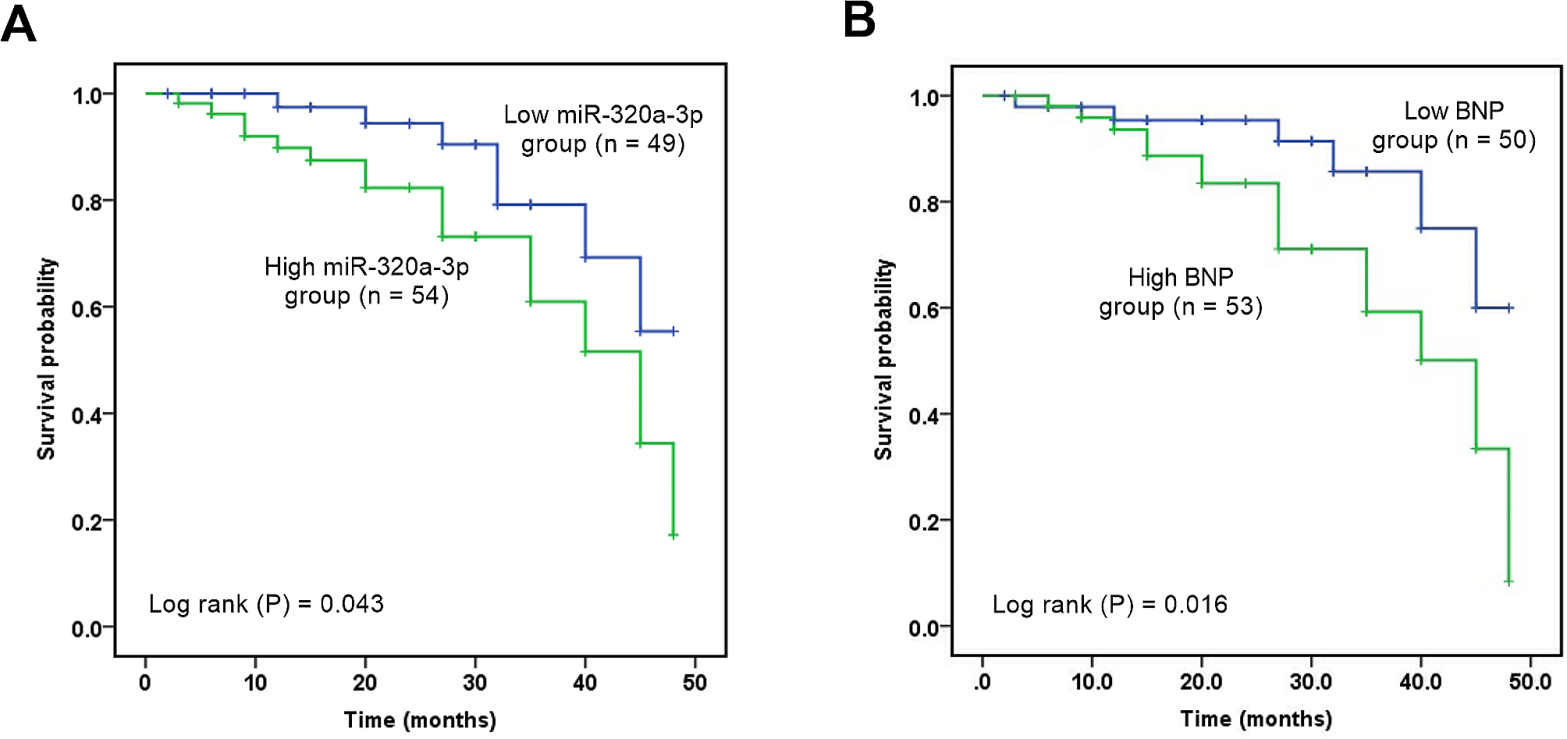
Kaplan-Meier survival curve. **(A)** Survival curve was grouped according to the mean expression level of miR-320a-3p. **(B)** Survival curve was grouped according to the mean level of BNP.

During the 4-year follow-up period, a total of 28 CHF patients (27.18%) died from cardiac causes. According to the performance of different groups, 7 patients in the low miR-320a-3p expression group died, and 21 patients in the high miR-320a-3p expression group died. In addition, according to the BNP grouping, 5 patients in the low BNP expression group died, while 23 patients in the high BNP expression group died. According to Kaplan-Meier curve, it was found that patients with high expression of miR-320a-3p or BNP had poor prognosis and survival (Fig. 3A-B, *P* < 0.05). Multivariate Cox regression showed that age (HR = 2.938, 95%CI = 1.326–7.707), BNP (HR = 3.608, 95%CI = 1.505–9.119) and miR-320a-3p (HR = 2.763, 95%CI = 1.089–7.031) were independent prognostic factors for 48-month survival in CHF patients (Table 2, *P* < 0.05).

**Table 2:** Multivariate Cox regression analysis for the overall survival of CHF patients

Characteristics	HR	95% CI	*P*
Age (years)	2.938	1.326–7.707	0.022
Gender (male/female)	1.229	0.541–3.116	0.558
BMI (kg/m^2^)	0.907	0.392–2.097	0.819
TC (mmol/L)	1.213	0.552–2.666	0.631
TG (mmol/L)	1.376	0.573–3.302	0.475
LDL-C (mmol/L)	1.367	0.584–3.201	0.471
HDL-C (mmol/L)	0.551	0.239–1.269	0.162
BNP (pg/mL)	3.608	1.505–9.119	0.009
LVEF (%)	1.234	0.556–2.740	0.606
Smoking history (yes/no)	0.955	0.411–2.217	0.904
Drinking history (yes/no)	1.074	0.454–2.537	0.871
Hypertension (yes/no)	1.902	0.690–5.246	0.214
Diabetes (yes/no)	1.787	0.729–4.378	0.204
miR-320a-3p	2.763	1.089–7.031	0.031

Abbreviations: CHF, chronic heart failure; BMI, body mass index; TC, total cholesterol; TG, triglyceride; LDL-C, low-density lipoprotein cholesterol; HDL-C, high-density lipoprotein cholesterol; BNP, brain natriuretic peptide; LVEF, left ventricular ejection fraction. *P* < 0.05 stands for significant difference

### Possible target gene of miR-320a-3p

**Fig. 4:**
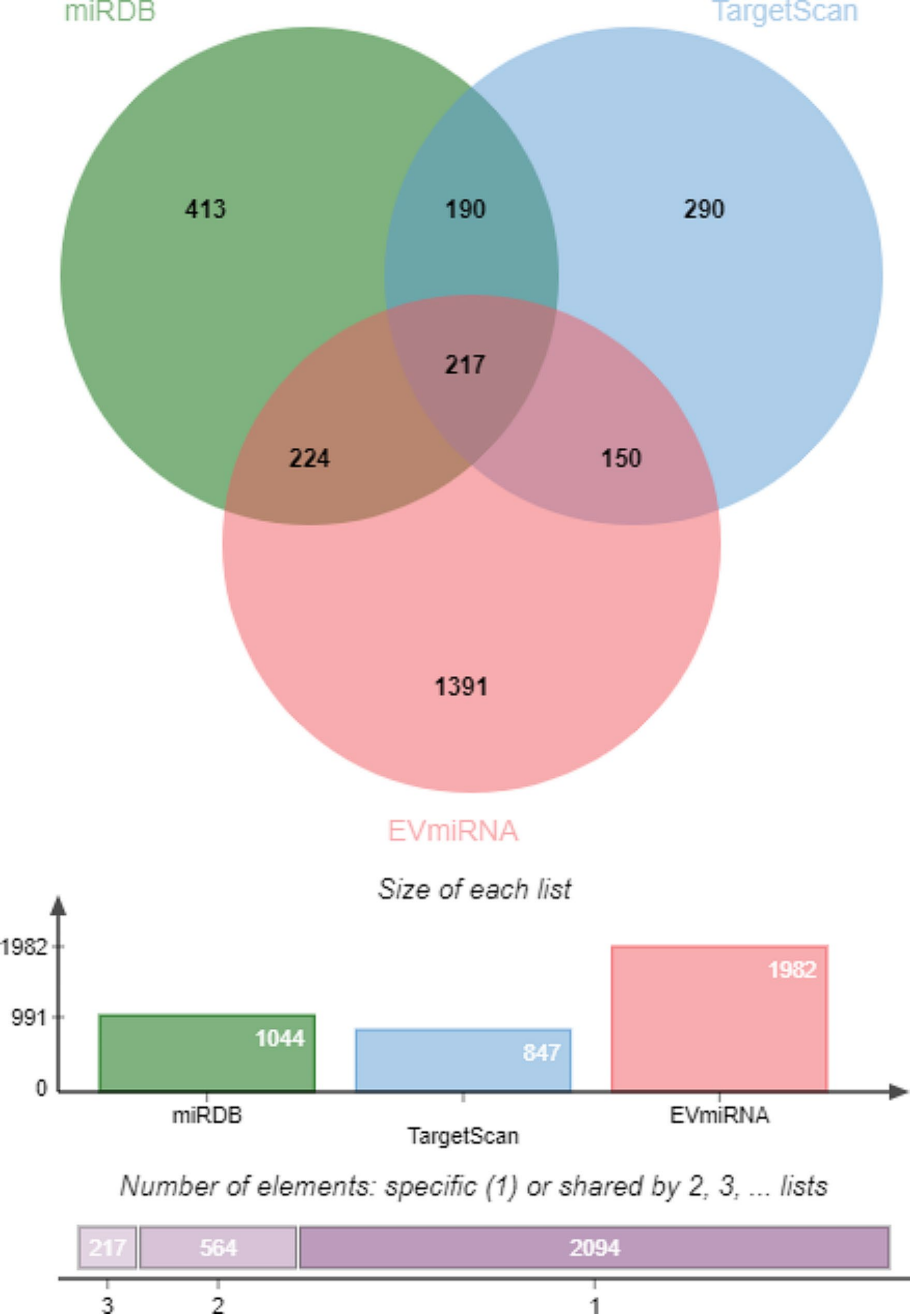
Venn diagram of the target genes of miR-320a-3p

As shown in Fig. 4, the miRDB database predicted 1044 target genes of miR-320a-3p, the TargetScan database predicted 847 target genes, and the EVmiRNA predicted 1982 target genes, with 217 target genes supported by all three databases. The specific names of these 217 target genes are summarized in Table 3.

**Table 3:** The target genes of miR-320a-3p predicted by TargetScan, miRBD and EVmiRNA

SH2B3	PCDHA11	TUSC3	HSPH1	CKAP5	ZC3H12C	RAPGEFL1
KITLG	PCDHA3	DTNA	KLHL15	FANCF	AZIN1	CREBRF
GPBP1	LMO3	ZNF652	FAM117B	E2F1	TIMM8B	ADAM10
CNKSR2	RASA1	GTPBP2	PRPS1	MAP1LC3B	ZBTB10	VLDLR
SDHD	HSPA4	AGFG1	PHC1	PPP2R2C	FBXO28	FAM49B
TFAP2B	PCDHA4	PFKM	IRF6	INO80D	MITF	CLCN5
NXT2	PCDHA1	SEMA3A	EXO1	LOXL3	HERPUD1	MIER1
BLCAP	PBX3	FAM160B1	RPL15	ZNF516	ALKBH5	TPD52
DCP1A	PCDHA7	KCNS3	KIAA1324	HOXA10	GLIS3	ANKRD52
NUFIP2	PCDHA2	MAPK8IP3	SLC10A3	DAZAP1	STAG2	CREB3L2
ZNF281	SGCB	COPS2	BMI1	DESI2	PCSK6	MSI2
MLLT3	PCDHA12	AGPS	STAT4	YIPF6	GOLGA3	MIER3
CPD	NPAS2	VDAC1	AP3M1	EIF3J	BVES	KDSR
ARPP19	ESRRG	IDE	HLCS	ULK1	CRKL	SCN1A
MTDH	PBX1	PCDHA9	STARD4	CHIC1	WLS	RANBP2
HELZ	BRWD3	SATB2	SOX4	NFIA	PCSK1	SLC10A7
DHDDS	GNS	RAB18	PTEN	MN1	AKT3	TNKS2
CDK13	CYLD	TSC1	ETFA	EIF2B1	VIM	DNAJA2
CDK6	PCDH19	GXYLT1	TPD52L2	TSC22D4	ZIC3	SF3B3
ARFIP1	GSPT1	BMPR1A	RAD51	SERINC3	ANKH	FOXM1
ENTPD4	TRIAP1	NAP1L5	HNRNPF	RRP1B	SORCS1	DNAJC3
PCDHA8	EOGT	ATL3	NCAPD3	MAK16	DAG1	RUNX1T1
PCDHAC1	RNF185	LRP6	DMD	TPM3	ATRX	DENND5B
RIT1	TMEM64	ARMCX2	RAP1A	PSMF1	SMG7	MAPK1
PCDHAC2	GNAI1	MXI1	NRK	CELSR3	ENPEP	ACVR2B
PCDHA13	DLX1	KLHL36	RGS9BP	PRKAG2	ZFP91	TMEM106B
PCDHA10	KLF13	FLRT3	BANP	UBE2Z	PTPRB	THUMPD3
PCDHA5	NRP1	CDH2	SIN3A	SLC6A8	PDS5A	HIC2
RAI2	ETV1	SOBP	SYNGR2	POLR1C	AKAP2	PIK3R1
PCDHA6	IGF2BP3	Section 63	RAB3GAP2	XIAP	DR1	NR3C1
FUS	DAB2	TMEM123	ARFGEF2	RAB11A	CISD2	FKBP5

### GO analysis and KEGG pathway analysis of miR-320a-3p target genes

**Fig. 5:**
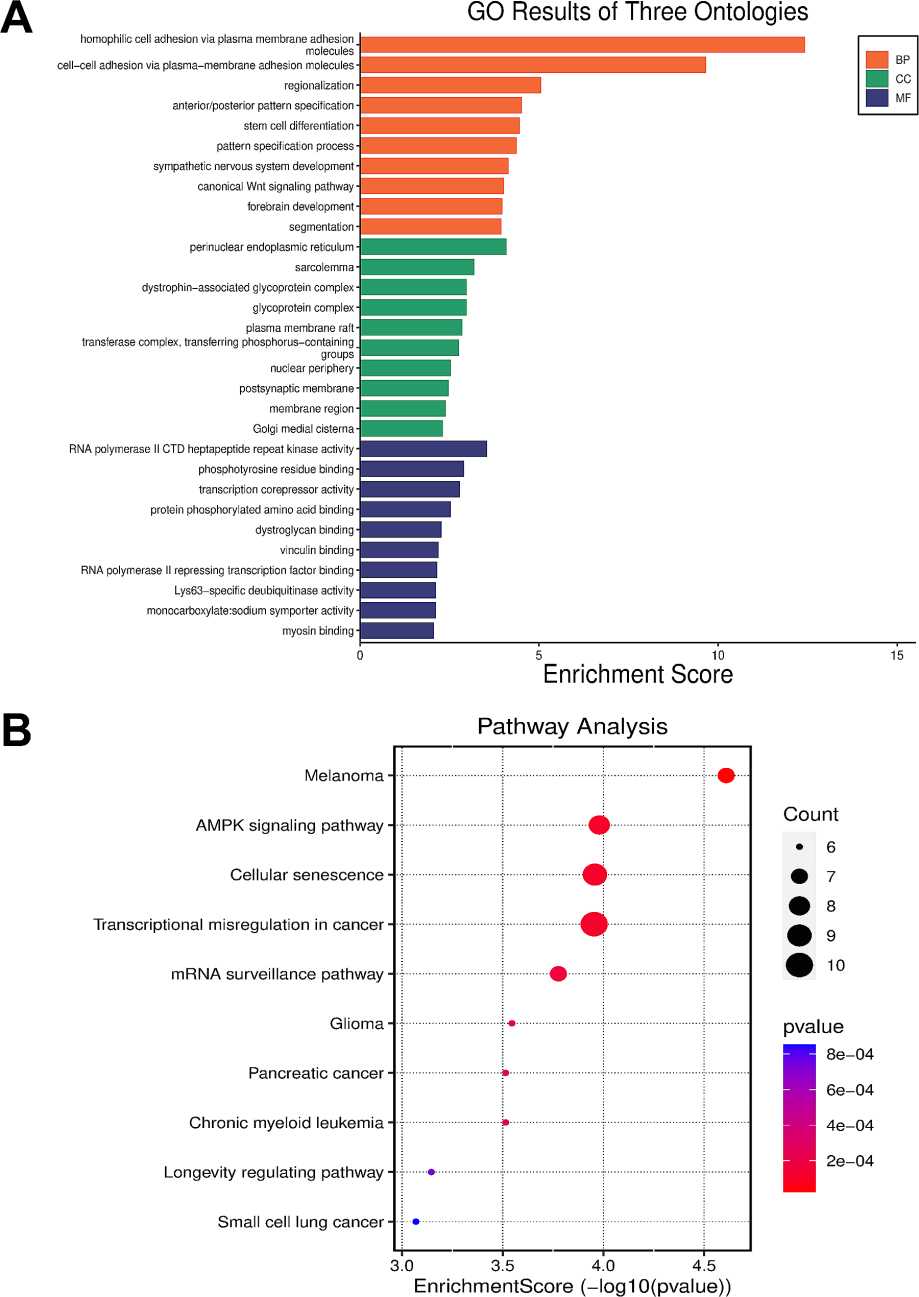
Bioinformatics analysis for target genes of miR-320a-3p. **(A)** Gene Ontology (GO) enrichment analysis. **(B)** Kyoto Encyclopedia of Genes and Genomes (KEGG) pathway analysis

GO enrichment analysis of the aforementioned 217 target genes showed that the target genes of miR-320a-3p were mainly located in the perinuclear endoplasmic reticulum and were mainly involved in molecular functions such as cell adhesion, stem cell differentiation, sympathetic nervous system development, regionalization, and Wnt signaling pathway. In addition, these target genes are involved in a variety of biological processes, including regulation of RNA polymerase activity and phosphotyrosine residue binding (Fig. 5A, *P* < 0.05). As shown in Fig. 5B, in KEGG analysis, target genes were mainly related to AMPK signaling pathway, cell aging, cancer-related pathways, autophagy, TNF signaling pathway, mTOR signaling pathway, insulin signaling pathway, hypertrophic cardiomyopathy, etc. AMPK, TNF, mTOR and insulin signaling pathways are known to be related to the pathogenesis of CHF.

## Discussion

In the present study, the miR-320a-3p level was increased in CHF patients and gradually upregulated with the increase of NYHA grading. MiR-320a-3p has shown certain clinical diagnostic value for CHF, and high expression of miR-320a-3p predicts poor prognosis of CHF patients. In addition, bioinformatics analysis exposed that the downstream target genes of miR-320a-3p are mainly involved in the regulation of AMPK, mTOR and TNF signaling pathways, all of which are related to the mechanism of CHF.

Among many miRNAs, the focus of this study is miR-320a-3p, a well-known miRNA, which is abnormally expressed in many cancers, such as lung cancer [17], breast cancer [18]. Previous studies have shown that miR-320a-3p levels in circulating plasma at baseline in non-survivors of cardiogenic shock are elevated and correlated with markers of hypoperfusion [19]. Second, Burns et al. found in a study on adverse drug reactions that miR-320a-3p was enhanced in the serum of patients with clozapine-induced cardiotoxicity [20]. These reports suggest that miR-320a-3p is abnormally expressed in heart-related diseases and may play a potential role in the progression of CHF. As demonstrated in the results of this study, compared with the control group without CHF, the serum miR-320a-3p level in patients with CHF increased. Studies have shown that the lower the LVEF, the higher the systolic blood pressure, the more serious the impairment of ventricular systolic function, and the more obvious the ventricular remodeling [21]. BNP is mainly stored in the ventricular muscle, and its secretion varies with the level of ventricular filling pressure. In patients with heart failure, the secretion of BNP in the heart muscle increases due to increased ventricular wall tension. Plasma BNP levels were positively correlated with the severity of heart failure [22]. In this study, the level of miR-320a-3p was positively correlated with BNP and negatively correlated with LVEF. To estimate the clinical value of abnormal miR-320a-3p in CHF, we evaluated the diagnostic and prognostic value of miR-320a-3p. The results showed that the AUC of miR-320a-3p was 0.866, which showed high diagnostic accuracy for CHF and control population. Meanwhile, survival analysis showed that high expression of miR-320a-3p was interconnected with increased mortality in 4-year CHF patients, which could be used as an independent prognostic indicator of CHF. BNP has been recommended by foreign guidelines as a classic indicator of HF clinical diagnosis and efficacy evaluation and is a relatively ideal HF biomarker [23]. BNP is mainly secreted by ventricular myocytes and has strong diuretic, natriuretic, vasodilator, anti-myocardial fibrosis and other effects [24]. In the clinical diagnosis of HF, when BNP detection is negative, it can be used to exclude HF. However, when BNP detection is positive, the presence of some interfering factors complicates its measurement. Potential factors for BNP positivity include age, weight, medicine, and kidney function. For example, plasma BNP levels can rise significantly in patients with kidney failure. In this situation, some other markers are needed clinically to assist the diagnosis of HF. As shown in this study, miR-320a-3p is significantly increased in patients with CHF and showed clinical significance for the diagnosis and prognosis of CHF. Therefore, it has the potential to be a diagnostic and prognostic marker for CHF.

CHF is an incurable disease, and the current treatment aims to prevent and delay the development of the disease, relieve clinical symptoms, reduce mortality and improve prognosis [25]. Previous studies have shown that miR-320a aggravates atherosclerosis by inhibiting RGS5 and promoting cell viability, migration, and proliferation of ox-LDL-induced VSMCs [26]. However, the promotion of CHF development by miR-320a-3p through regulation of downstream targets remains largely unknown. In this study, 217 downstream target genes of miR-320a-3p were predicted through the 3 online database, and further bioinformatics analysis showed that these target genes are enriched in various biological processes such as stem cell differentiation, neural development, and regionalization. In addition, these target genes mainly exist in the mTOR/AMPK/TNF/ insulin signaling pathways. Wang et al. reported that miR-320a accelerated the proliferation of myocardial fibroblasts by regulating the mTOR signaling pathway in HEH2 cells [27]. Li et al. claimed that miR-320a-3p prevented the development of medial arterial calcification through the AMPK/mTOR autophagy pathway [28]. After searching the published literature, we did find that miR-320a-3p is associated with predicted target genes and associated signaling pathways in animal or cellular models.

However, this study has the following shortcomings: first, this is a single-center clinical study with a small sample size, which may lead to selection bias. Therefore, it is necessary to expand the sample size to further support the conclusions of this study. Secondly, this study did not dynamically observe the changes in the expression level of miR-320a-3p in the blood of CHF patients. For example, with the improvement of cardiac function, the change trend of circulating miR-320a-3p expression level in patients is still unknown. In addition, this study did not further explore the possible mechanism of miR-320a-3p in CHF, so further exploration should be carried out in future studies.

In conclusion, this study suggests that miR-320a-3p expression level is up-regulated in patients with CHF, and abnormal levels of miR-320a-3p in CHF may be an effective biomarker for diagnosis and survival prognosis of CHF. In addition, the results of bioinformatics studies preliminarily clarified the target genes and related signaling pathways of miR-320a-3p, providing a theoretical basis for further research on the role of miR-320a-3p in CHF.

## Data Availability

Corresponding authors may provide data and materials.
